# Heat transfer through hydrogenated graphene superlattice nanoribbons: a computational study

**DOI:** 10.1038/s41598-022-12168-7

**Published:** 2022-05-13

**Authors:** Maryam Zarghami Dehaghani, Sajjad Habibzadeh, Omid Farzadian, Konstantinos V. Kostas, Mohammad Reza Saeb, Christos Spitas, Amin Hamed Mashhadzadeh

**Affiliations:** 1 School of Chemical Engineering, College of Engineering, University of Tehran, Tehran, Iran; 2 Department of Chemical Engineering, Amirkabir University of Technology (Tehran Polytechnic), Tehran, Iran; 3 Mechanical and Aerospace Engineering, School of Engineering and Digital Sciences, Nazarbayev University, 010000 Nur-Sultan, Kazakhstan; 4 Department of Polymer Technology, Faculty of Chemistry, Gdańsk University of Technology, G. Narutowicza 11/12, 80-233 Gdańsk, Poland

**Keywords:** Materials science, Theory and computation, Computational methods

## Abstract

Optimization of thermal conductivity of nanomaterials enables the fabrication of tailor-made nanodevices for thermoelectric applications. Superlattice nanostructures are correspondingly introduced to minimize the thermal conductivity of nanomaterials. Herein we computationally estimate the effect of total length and superlattice period ($l_{p}$) on the thermal conductivity of graphene/graphane superlattice nanoribbons using molecular dynamics simulation. The intrinsic thermal conductivity ($\kappa_{\infty }$) is demonstrated to be dependent on $l_{p}$. The $\kappa_{\infty }$ of the superlattice, nanoribbons decreased by approximately 96% and 88% compared to that of pristine graphene and graphane, respectively. By modifying the overall length of the developed structure, we identified the ballistic-diffusive transition regime at 120 nm. Further study of the superlattice periods yielded a minimal thermal conductivity value of 144 W m^−1^ k^−1^ at $l_{p}$ = 3.4 nm. This superlattice characteristic is connected to the phonon coherent length, specifically, the length of the turning point at which the wave-like behavior of phonons starts to dominate the particle-like behavior. Our results highlight a roadmap for thermal conductivity value control via appropriate adjustments of the superlattice period.

## Introduction

From the first exfoliation of graphite to graphene synthesis, two-dimensional (2D) nanomaterials have become worth of profound consideration. Graphene, sp^2^-hybridized carbon known for its honeycomb crystal lattice, shows an outstanding electrical conductivity^1^, Young's modulus^2^, thermal conductivity^3,4^, adsorption capacity^5^, and surface area^6^, which are needed for various biological, environmental, and engineering applications^7–12^. However, the semiconductor and transistor applications of graphene are challenging due to its zero bandgap. Furthermore, thermal performance optimization of graphene, for thermoelectric energy conversion and optoelectronic devices, requires chemically-hybridized graphene with some other 2D nanomaterials, such as boron-carbide^13,14^, nitrogen-carbide^15^, graphane^16^, beryllium-oxide^17,18^, silicone-germanium^19^, or boron-nitride^20–25^. The resulting heterostructures are named hybrid superlattice nanosheets^26^.

Graphane, i.e., fully hydrogenated graphene possessing carbon atoms in the form of sp^3^ hybridization that create C–H bonds, is a semiconductor and insulator having a bandgap in the range of 3.5–4.4 eV^27^. Graphane can be found in different conformations; however, the so-called *chair* conformation, in which hydrogen atoms alternate their connection below and above the graphene sheet^28^, is known as the most stable one. Graphane structure was first experimentally reported in 2009 by Elia et al., obtained through graphene exposure to cold hydrogen plasma at a pressure of 0.1 mbar^29^. Then, properties of pristine or hybrid forms of graphane were reported in the pertinent literature. Namely, the heat capacity of graphane was estimated by 29*.*32 ± 0.23 J mol^−1^ K^-1^, as predicted using molecular dynamics (MD) simulation by Neek-Amal^30^. The 2D Young^'^s modulus of graphane was computed to be 245 N m^−1^ employing density functional theory (DFT), smaller than the value of Young^'^s modulus reported for 340 ± 50 N m^−1^ graphene^31^.

Pei et al.^32^ reported that complete hydrogenation of graphene reduces the thermal conductivity by about 70–80% due to the softening of G-band phonon modes upon the transition of sp2 sp^3^ bond. Rajabpour et al.^33^ found the thermal rectification and interface thermal resistance of hybrid graphene-graphane nanoribbons, demonstrating the potential usage of this hybrid nanoribbon as a promising thermal rectifier. The thermal conductivity of multilayer graphene/graphane/graphene heterostructure nanoribbons was also studied using MD simulation by Kim et al.^34^. They reported that the chemical integration of graphene with graphane creates heteronanosheets where a decrease in the thermal conductivity of graphene to a desirable level by 96% can be obtained. These cases mentioned above indicate the effort of researchers to manipulate the thermal conductivity of such structures towards the fabrication of nanodevices with controlled thermal conductivity. In this regard, superlattice nanostructures are introduced for minimizing thermal conductivity. Such superlattice structure consists of a repeating unit containing different materials with a specified periodic length ($l_{p}$, superlattice period). The fabricated superlattice has a lower thermal conductivity value than its constituent components. This minimal thermal conductivity phenomenon stems from the competitive nature of wave-like (coherent) and particle-like (incoherent) modes of phonon thermal transport. Namely, as the value of $l_{p}$ is larger than the phonon coherence length, the incoherent mode of thermal transport dominates the coherent mode such that the minimal thermal conductivity occurs at the point of coherent-incoherent transition^35^.

Several theoretical research works have been performed to unravel the thermal transport in the superlattice nanostructures. For example, the dependency of thermal conductivity of graphene-hBN superlattice nanoribbons on the superlattice period as well as the total length, based on MD simulation, were examined^35^. It was found that the thermal conductivity of a nanoribbon with a superlattice period of 3.43 nm was equal to 89 W m^−1^ K^−1^, which is lower than the thermal conductivity of either graphene or hBN nanoribbons. Moreover, the effective phonon mean free path (MFP) was estimated to be minimum (32 nm) for the same superlattice period. It was found that the suppression of coherent phonon thermal transport is achieved by increasing the number of interfaces per unit cell. Correspondingly, the Fibonacci expression is enlarged due to the growth of the hindering phonon coherence along the superlattice nanoribbon^36^. Wang et al.^37^ resulted in infinite thermal conductivities of 16.08, 15.95, 5.60 W m^−1^ K^−1^ for silicene, pure germanene, and silicene-germanene superlattice nanoribbon, respectively. The dependencies of thermal conductivity on the total length, temperature, and the temperature difference between hot and cold baths for the C_3_N/C_2_N superlattice nanosheets were studied by Razzaghi et al*.* through the MD simulation^37^. They obtained the values of 23.2 W m^−1^ K^−1^, and 24.7 nm for the minimum thermal conductivity and the phonon mean free path, respectively, at a superlattice period of 5.2 nm. Moreover, they reported that at a total length larger than 80 nm, the scattering of low-wavelength or high-frequency phonons at interfaces occurs. By contrast, at shorter lengths, the wave interference causes a reduction in the amount of thermal conductivity. According to the performed theoretical studies, it can be understood that the minimum infinite thermal conductivity of superlattice nanoribbons is controlled by the superlattice period and the constituent elements of superlattice structures.

In the present work, we evaluate the thermal properties of a series of lateral graphene/graphane superlattice nanoribbons using MD simulations. The study was conducted with various total lengths and superlattice periods, at a mean reference temperature of 300 K and a temperature difference (ΔT) of 40 K between the two sides. Firstly, we verified the effect of the graphene/graphane sample's total length on the thermal conductivity of the superlattice nanoribbon with different superlattice periods ($l_{p}$). Subsequently, the effect of superlattice periods on the intrinsic thermal conductivity and the effective phonon MFP of graphene/graphene superlattice nanoribbon were explored.

## Simulation method

**Figure 1:**
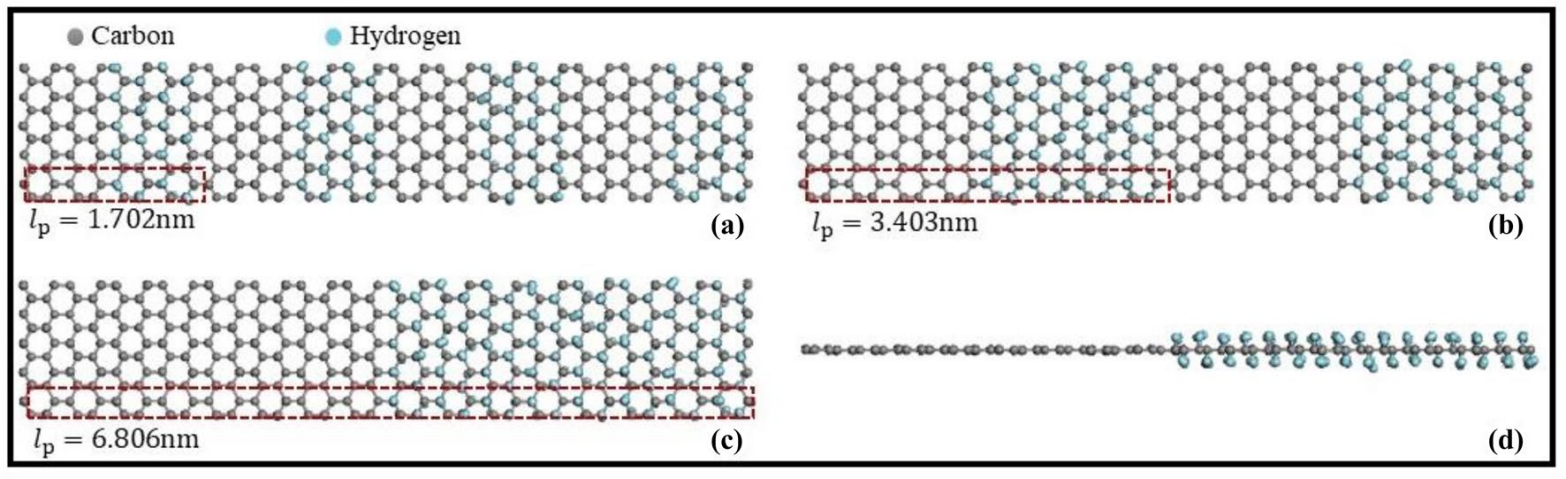
A unit cell of graphene/graphane superlattice nanoribbons having various superlattice periods (**a**) $l_{p}$ = 1.702 nm, (**b**) $l_{p}$ = 3.403 nm, and (**c**) $l_{p}$ = 6.806 nm. (**d**) front view of the unit cell depicted in (**c**).

In the current research, MD simulations conducted with the help of the open-source software package LAMMPS (Large-Scale Atomic/Molecular Massively parallel Simulator^38^) were used to investigate the thermal properties of graphene/graphane superlattice nanoribbons having various superlattice periods ($l_{p}$ = 1.702, 3.403, and 6.806 nm). The carbon–carbon and carbon–hydrogen bonding interactions were modeled using the adaptive intermolecular reactive empirical bond order (AIREBO) potential^39^ with a time-step of 0.25 fs. The monolayer superlattice nanoribbons were formed by repeating unit cells of graphene and graphane of the same size, i.e., equal to the superlattice period ($l_{p}$), as shown in Fig. 1. The width of all nanoribbons is 8 nm and periodic boundary conditions were considered in X and Y (in-plane) directions.

To correctly estimate the considered superlattice's thermal properties, several MD stages must be performed. The employed procedure within our numerical simulations comprises the following steps:

1. Firstly, energy minimization is performed for repositioning atoms in the constructed nanoribbon.
2. Secondly, an N.P.T. ensemble (constant atom number, pressure, and temperature) with the use of the Nosé-Hoover thermostat and barostat^40^ is applied to reach zero pressure and a temperature of 300 K in 250 ps.
3. Next, the system was equilibrated via the application of an N.V.T. ensemble (constant atom number, volume, and energy) for 1 ns at 300 K.
4. Finally, the thermal properties of superlattice nanoribbons are computed using the following setup: nanoribbon models are divided into 30 slabs along the X-direction. Atoms present in the left and right edges of superlattice nanoribbon are fixed. The NVE ensemble is applied to the cold and hot boundary slabs region. By applying the N.V.T. ensemble (Nose–Hoover thermostat method), the temperature at the hot and cold slabs is defined as $T + \Delta T/2$ and $T - \Delta T/2$, respectively, where *T* is the mean reference temperature. Finally, the following expression is used for heat flux ($J_{x}$) along the X direction^41^:
  1 $$J_{x} = \frac{{{\raise0.7ex\hbox{${dE}$} \!\mathord{\left/ {\vphantom {{dE} {dt}}}\right.\kern-\nulldelimiterspace} \!\lower0.7ex\hbox{${dt}$}}}}{{A_{c} }},$$
  $A_{c}$ refers to the cross-sectional area of the nanoribbons (0.34 × 8 nm^2^), t is the simulation time, and $E$ corresponds to accumulated energy.

After reaching steady-state conditions, the thermal conductivity for a nanoribbon of length L is calculated from Fourier law as follows:

2 $$K_{L} = \frac{{\left\langle {J_{x} } \right\rangle }}{{\left\langle {\nabla_{x} T} \right\rangle }}$$

$\left\langle . \right\rangle$ refers to time averages and $\nabla_{x} T$ denotes the temperature gradient in the direction of heat flow.

## Results and discussion

**Figure 2:**
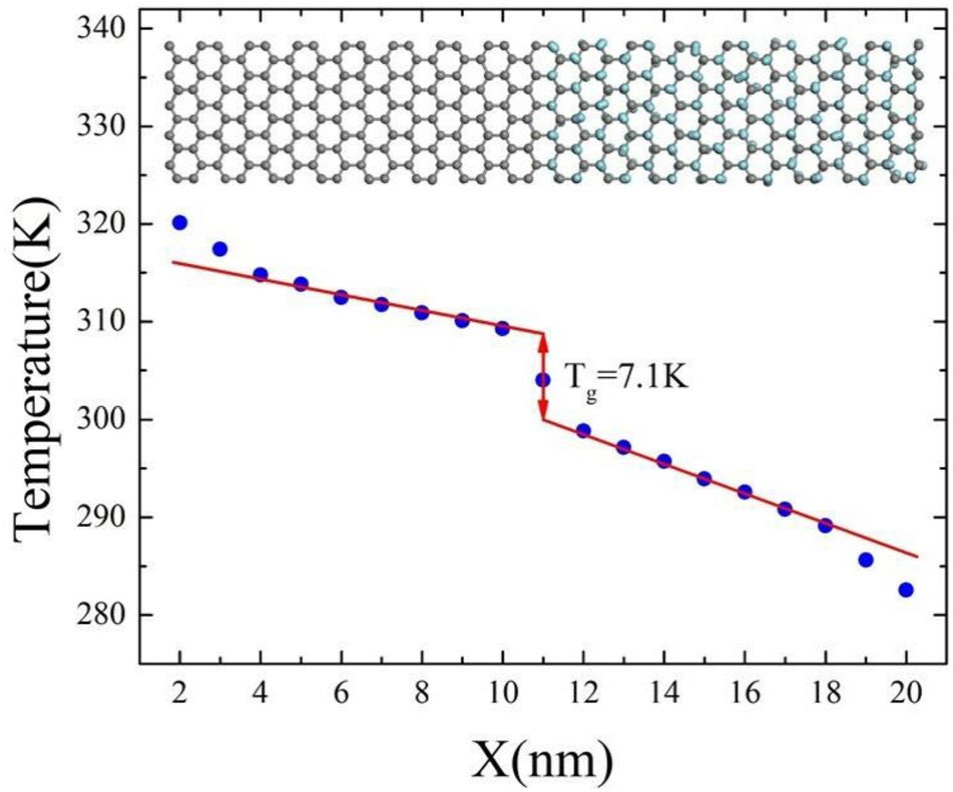
Steady-state: one-dimensional temperature profile for graphene/graphane hybrid nanoribbon with a length of 20.42 nm at T = 300 K and ΔT = 40 K.

The superlattice hybrid nanoribbons inevitably contain the interfaces at which the two alternating media meet each other. The presence of these interfaces and their total number affect heat transfer in these nanoribbons. Firstly, we investigate the effect of an individual interface on heat transfer. Figure 2 depicts the temperature profile of a single unit, graphene/graphane hybrid nanoribbon, with a length of 20.42 nm at room temperature and a temperature difference of 40 K. As we can observe in the same figure, the temperature profile exhibits a discontinuity at X = 11 nm, which coincides with the interface's position. The difference in atomic structures across the interface causes phonon scattering and, consequently, a temperature drop (T_g_) of 7.1 K. A similar behavior along the interface of a graphene-boron nitride heterostructure for the steady-state temperature is reported by Li et al.^42^.

**Figure 3:**
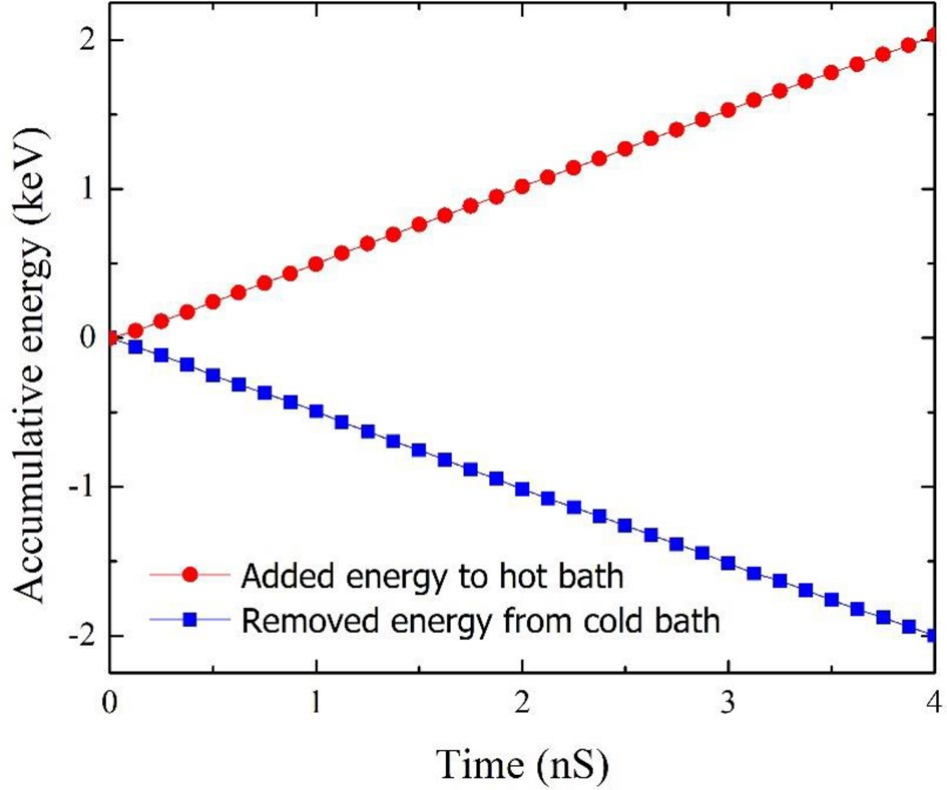
Accumulative energy changes in cold and hot slabs as a function of simulation time for graphene/graphane hybrid nanoribbon with a length of 20.42 nm at T = 300 K and ΔT = 40 K.

Figure 3 depicts the accumulated energy for the hot and cold slabs in the same graphene/graphane hybrid nanoribbon. Using Fig. 3 we can also compute the heat flux value ($\left| {\frac{dE}{{dt}}} \right| = 0.512 {\text{keV}}.{\text{ns}}^{ - 1}$) for the hot and cold slabs. Furthermore, this equality of the absolute amount of energy in the cold and hot baths is expected and aligned with the conservation of energy^43^.

The interfacial thermal resistance at the grain boundary (Kapitza resistance, $R_{k}$) can be calculated as follows^44^:

3 $$R_{k} = \frac{{T_{g} }}{{J_{x} }},$$

$T_{g}$ denotes the temperature drop at the interface (7.1 K) depicted in Fig. 2 and $J_{x}$ corresponds to heat flux obtained from Eq. (1). Therefore, the Kapitza resistance at the graphene-graphane interface is equal to 2.59 × 10^−11^ m^2^ K W^−1^.

### Thermal conductivity of graphene/graphane superlattice nanoribbon as a function of sample length

The effective thermal conductivity of a nanoribbon having a finite length *L*, under the simulation above conditions is calculated as follows^45^:

4 $$\frac{1}{{\kappa_{L} }} = \frac{1}{{\kappa_{\infty } }}\left( {1 + \frac{{\Lambda_{eff} }}{L}} \right),$$

**Figure 4:**
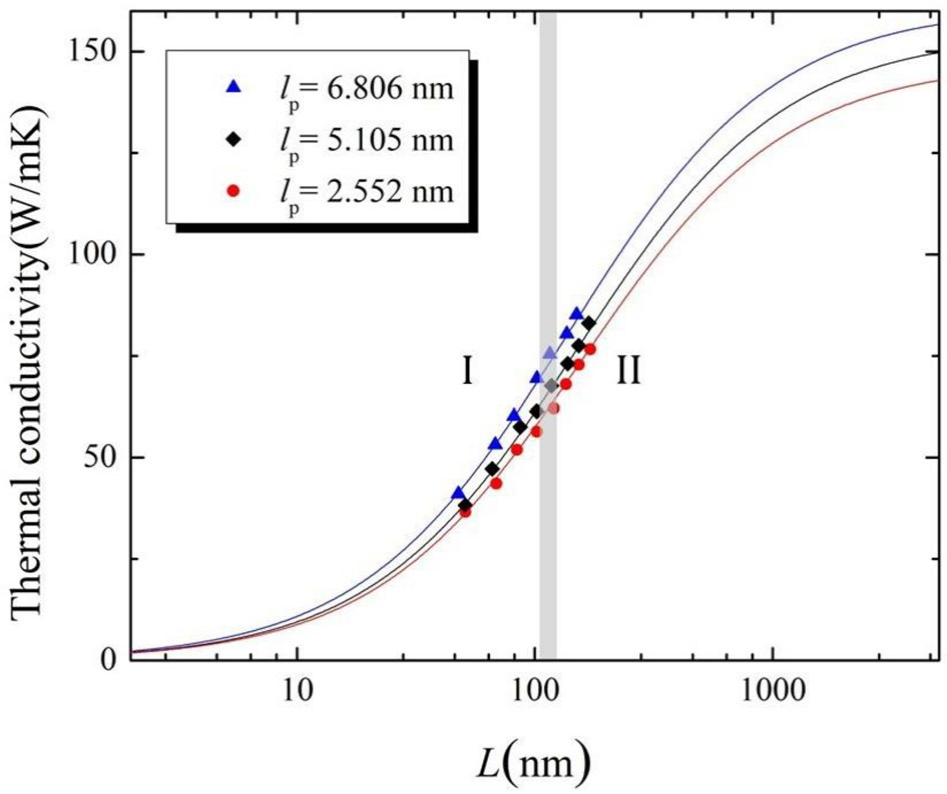
Thermal conductivity as a function of total length for graphene/graphane superlattice nanoribbons using various superlattice periods of $l_{p}$ = 2.552, $l_{p}$ = 5.105 nm, and $l_{p}$ = 6.806 nm at T = 300 K and ΔT = 40 K.

$\kappa_{\infty }$ is related to the intrinsic, or length-independent, the thermal conductivity of nanoribbon, and $\Lambda_{eff}$ corresponds to the effective phonon MFP. The effective phonon MFP corresponds to the length at which thermal conductivity is equal to half the value of the intrinsic thermal conductivity of the sample, i.e., if $\Lambda_{eff} = L$, then $\kappa = \frac{{\kappa_{\infty } }}{2}$. Figure 4 depicts thermal conductivity of graphene/graphane superlattice nanoribbons as a function of total length for three superlattice periods (6.806, 5.105, and 2.552 nm) at room temperature and a temperature difference of 40 K. Equation 4 was used to fit the data points and calculate $\kappa_{\infty }$ and the effective MFP As one may observe in Fig. 4, increasing the total length of the superlattice nanoribbon leads to an increase of thermal conductivity, as it is expected from Eq. (4). This positive correlation can be explained by the vanishing scattering for phonons with long wavelengths. However, after a certain length threshold, thermal conductivity increases slowly and reaches a plateau value which corresponds to $\kappa_{\infty }$. A similar positive correlation of thermal conductivity with sample's length was observed for BC_3_/C_3_N superlattice nanoribbons in the theoretical work performed by Mayelifartash et al.^46^. A linear behavior ($\kappa \propto L$) is exhibited for sample lengths above 50 nm and till approximately 500 nm. Two heat transport regimes (I and II) can be identified: region I, corresponding to the ballistic regime, exhibits phonon MFPs which are larger than the length of nanoribbon and phonons may travel at distances exceeding the coherence length. Region II is the diffusive regime where phonon MFPs are shorter than the total length and thermal conductivity loses gradually its dependence on nanoribbon's length. The gray overlay in Fig. 4, where phonon MFP and total length are of the same magnitude, depicts the ballistic-diffusive transition regime. See^47^ for more detail about the contribution of ballistic and diffusive components to the total thermal conductivity.

### Thermal conductivity and effective phonon MFP as a function of superlattice period

**Figure 5:**
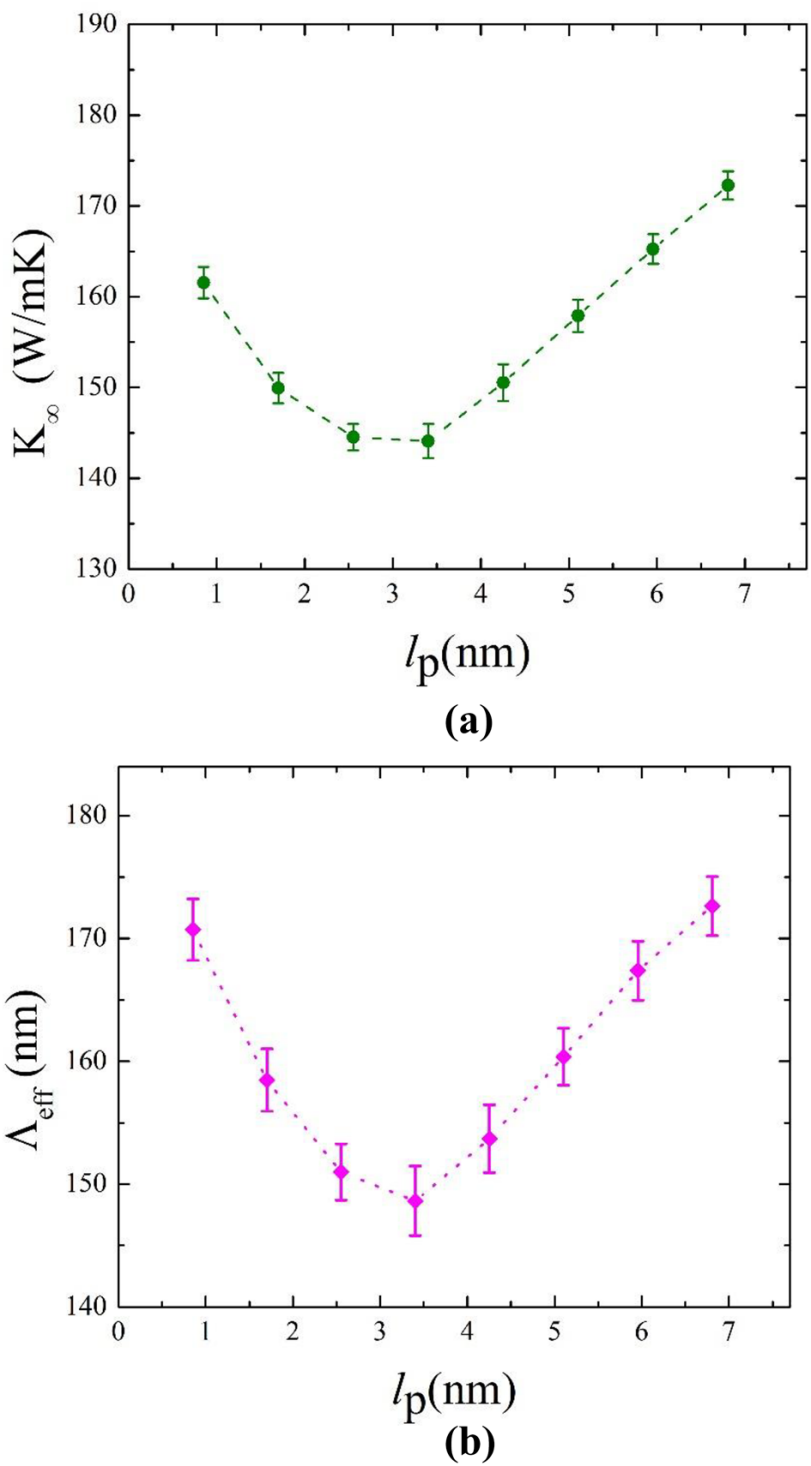
(**a**) Intrinsic thermal conductivity and (**b**) effective phonon MFP as functions of superlattice period for graphene/graphane superlattice nanoribbons at T = 300 K and ΔT = 40 K.

The dependency of the intrinsic thermal conductivity of superlattice nanoribbons on their period can be exploited when designing thermal superlattice nanodevices. Figure 5 demonstrates the dependence of thermal conductivity and effective phonon MFP on superlattice's period. We consider periods ranging from 0.85 to 6.8 nm at room temperature and a temperature difference of 40 K. As indicated in Fig. 5a, the intrinsic thermal conductivity values for all graphene/graphane superlattice periods are less than the thermal conductivity of either graphene or graphane^48^. Specifically, the thermal conductivity of the superlattice nanoribbon decreases by ≈96.4% and 88% compared to the thermal conductivity of pristine graphene and graphane, respectively. Another interesting observation in the same figure relates to the behavior of $\kappa_{\infty }$. Initially, the value of $\kappa_{\infty }$ decreases with increasing superlattice period values till it reaches a minimum value of 144 W m^−1^ k^−1^ at $l_{p}$ = 3.4 nm. Afterwards, a reverse trend is observed. A similar non-monotonic dependency of $\kappa_{\infty }$ on $l_{p}$ was also observed for hBN/graphene superlattice nanoribbons in^36,49^. Furthermore, Farzadian et al*.*^50^ reported a similar minimum thermal conductivity value of 155 W m^−1^ k^−1^ for graphene/phagraphane superlattice nanoribbons at a superlattice period 12.85 nm.

This minimum thermal conductivity occurs at the transition from coherent to incoherent phonon transport. Before reaching the minimum value of $\kappa_{\infty }$, the occurrence of Brillouin zone folding and band flattering as a result of phonon wave effect along with the modification of the bulk phonon dispersion relation causes the reduction of phonon group velocities and leads to decreasing thermal conductivity with increasing values of $l_{p}$^51^. After the minimum point, at which particle-like phonons scatter diffusively at the interfaces, increases in $l_{p}$ facilitate heat transfer, and subsequently thermal conductivity, as thermal resistors (media interfaces) decrease in number. Therefore, the minimum thermal conductivity happens at the length of the wave interference effects and the diffuse interface scattering overlap^52^.

For further elucidating the dependency trend of thermal conductivity on $l_{p}$, the concepts of effective phonon MFP ($\Lambda_{eff}$) and phonon coherent length are discussed. Effective MFP is defined as the average distance that phonon travels before scattering. Phonon coherent length at which the minimum thermal conductivity occurs corresponds to the length that wave-like behavior of phonons starts to become more significant than particle-like behavior^53^. As shown in Fig. 5, the phonon coherence length is estimated at 3.4 nm (42 times smaller than the minimum of $\Lambda_{eff}$ which coincides with the same value of the superlattice period). This means that MFP reaches distances much larger than the coherence length, which is also in agreement with the increasing trend in thermal conductivity depicted in Fig. 4.

The wave-like properties are dominant to the left of the minimum. So, phonons suffer a small influence on the interfaces, and transport is coherent. This explains the reduction of thermal conductivity when increasing $l_{p}$. To the right of the minimum, particle-like incoherent properties are dominant. Thus easing heat conduction due to the decreasing number of interfaces (thermal resistors) with $l_{p}$. Indeed, it has been shown that thermal conductivity decreases when the structure periodicity is dominated by wave interference effects and increases when it depends on diffuse interface scattering^47,54,55^.

**Figure 6:**
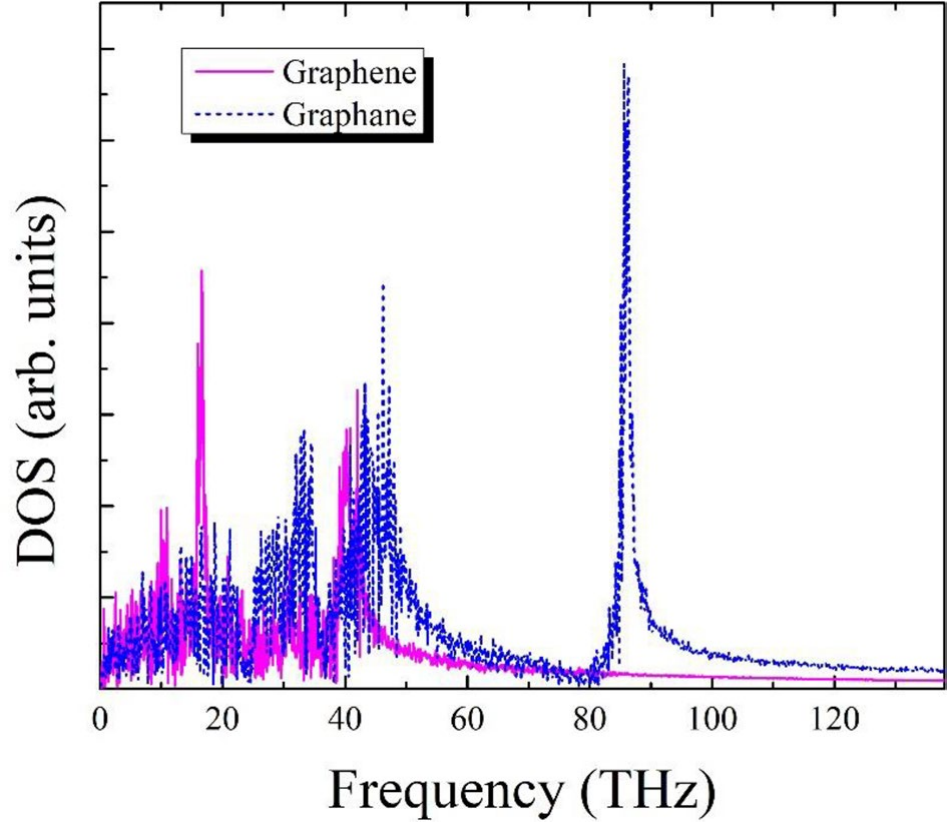
Phonon density of state (DOS) on two sides of graphene (left) /graphene (right) hybrid nanoribbon interface having a length of 20.42 nm at T = 300 K and ΔT = 40 K.

As a final stage in this research to achieve a deeper understanding of the interface effect in superlattice nanoribbons, we calculate the phonon density of state (DOS) for two groups of atoms at the left and right sides of the graphene/graphane interface; see Fig. 6. DOS of phonons on each side is obtained by calculating the Fourier transform of the velocity autocorrelation function as shown below:

5 $${\text{DOS}}\left( \omega \right) = \mathop \int \limits_{0}^{\infty } {\varvec{v}}\left( 0 \right) \cdot {\varvec{v}}\left( t \right)dt,$$

$\omega$ is the frequency and ${\text{DOS }}\left( \omega \right)$ denotes the density of states at $\omega$. Symbols ${\varvec{v}}\left( 0 \right)$ and ${\varvec{v}}\left( t \right)$ correspond to the velocity at time zero and $t$, respectively. Finally, $\left\langle . \right\rangle$ represents the average per atom over time.

As can be observed, there is a substantial mismatch between the left and right spectrum. This asymmetry of phonon spectra unravels the interfacial thermal resistance and the asymmetrical phonon scattering at the graphene/graphene interface.

## Conclusion

In this study, MD simulations were applied to study the effect of the total length on the thermal conductivity of graphene/graphane superlattice nanoribbons. At the same time, various superlattice periods ($l_{p}$) were examined and their effect on the intrinsic thermal conductivity ($\kappa_{\infty }$) was established. Furthermore, the behavior pattern for the intrinsic thermal conductivity and the effective phonon MFP of graphene/graphene superlattice nanoribbon with increasing values of the period $(l_{p} )$ was identified and studied.

In terms of the sample's total length effect, it was observed that an increase in total length leads to elevated thermal conductivity values. However, thermal conductivity at considerable lengths becomes independent from the length and reaches a plateau value. Moreover, two regions of heat transport were identified based on the sample's total length. The turning point between these two regions occurs at the neighborhood of 120 nm, which corresponds to the ballistic-diffusive transition regime. With regards to superlattice period variation, a minimum thermal conductivity of 144 W m^−1^ k^−1^ is observed at $l_{p}$ = 3.4 nm. This superlattice period signifies phonon coherent length and corresponds to the length at which wave-like behavior of phonons starts to dominate particle-like behavior. Our results indicate that introducing graphane stripes, at appropriate regular intervals, to a graphene sheet permits the control of the resulting hybrid structure's thermal conductivity. Such manipulation capacity of thermal conductivity values is a promising tool for nanoelectronics.
